# Association of Genetic vs Environmental Factors in Swedish Adoptees With Clinically Significant Tinnitus

**DOI:** 10.1001/jamaoto.2018.3852

**Published:** 2019-01-17

**Authors:** Christopher R. Cederroth, MirNabi PirouziFard, Natalia Trpchevska, Esma Idrizbegovic, Barbara Canlon, Jan Sundquist, Kristina Sundquist, Bengt Zöller

**Affiliations:** 1 Department of Physiology and Pharmacology, Karolinska Institutet, Stockholm, Sweden; 2 Centre for Primary Health Care Research, Department of Clinical Sciences, Malmö, Lund University, Malmö, Sweden; 3 Hörsel-och Balanskliniken, Karolinska Universitetssjukhuset, Stockholm, Sweden

## Abstract

**Importance:**

No effective treatments are currently available for severe tinnitus, which affects 1% of the population and lowers the quality of life. The factors that contribute to the transition from mild to severe tinnitus are poorly known. Before performing genetic analyses and determining the mechanisms involved in the development of severe tinnitus, its heritability needs to be determined.

**Objectives:**

To examine whether clinically significant tinnitus is associated with genetic factors and to evaluate the genetic risk in the transmission of tinnitus using adoptees.

**Design, Setting, and Participants:**

Data from adoptees and their biological and adoptive parents from Swedish nationwide registers were collected from January 1, 1964, to December 31, 2015, and used to separate genetic from environmental factors in familial clustering. In all, 11 060 adoptees, 19 015 adoptive parents, and 17 025 biological parents were investigated. The study used a cohort design and a case-control approach to study genetic and nongenetic factors in tinnitus among adoptees.

**Main Outcomes and Measures:**

The primary outcome was odds ratio (OR) of tinnitus in adoptees with at least 1 affected biological parent compared with adoptees without any affected biological parent using logistic regression. The secondary outcome was OR in adoptees with at least 1 affected adoptive parent compared with adoptees without any affected adoptive parent.

**Results:**

A total of 1029 patients (440 [42.8%] male; mean [SD] age, 62 [14] years) with tinnitus were identified. The prevalence of diagnosed tinnitus was 2.2%. The OR for tinnitus was 2.22 for adoptees (95% CI, 1.03-4.81) of biological parents diagnosed with tinnitus, whereas the OR was 1.00 (95% CI, 0.43-2.32) for adoptees from adoptive parents diagnosed with tinnitus. Mean (SE) heritability determined using tetrachoric correlations was 31% (14%).

**Conclusions and Relevance:**

The findings suggest that genetic factors are associated with the familial clustering of clinically significant tinnitus with no shared-environment association, revealing that the transition from negligible to severe tinnitus may be associated with genetic factors. These findings may provide insight for future genetic analyses that focus on severe tinnitus.

## Key Points

**Question:**

Is clinically significant tinnitus associated with genetic factors?

**Findings:**

In this study of national registry data from 11 060 adoptees, 19 015 adoptive parents, and 17 025 biological parents, a heritability of 32% and no association of shared environment with the transmission of tinnitus were found.

**Meaning:**

The present study suggests that genetic factors are associated with the familial clustering of severe tinnitus.

## Teaser

This study uses Swedish national registry data to assess the association between genetic vs environmental factors and risk of tinnitus among adoptees.

## Introduction

Tinnitus is one of the most prevalent and distressing symptoms associated with hearing loss. It is defined by the perception of sounds despite their physical absence (the phantom perception of sounds).^1^ This auditory condition affects more than 15% of the population worldwide (an estimated 70 million people in Europe). In 1 to 2 of 10 people, tinnitus becomes a chronic bothersome and incapacitating symptom,^2,3^ with highly unmet clinical needs.^4^ The psychological consequences of tinnitus affect the economy by influencing work and sleep and increasing the risk of sick leave and disability pension.^5^

Tinnitus is a complex and heterogeneous auditory dysfunction with numerous causes and phenotypes.^6^ It is frequently associated with hearing problems and noise exposure,^7^ but it also occurs in humans with normal hearing.^8^ The current models stipulate that tinnitus mimics the processes of phantom limb perception,^9^ whereby the loss of sensory input (most often by sensory deafferentation) leads to compensatory mechanisms in the brain that cause the false sensation of a missing limb or sounds.^8^ This phenomenon of maladaptive plasticity in the presence of deafferentation appears as a common denominator of most forms of phantom percepts in the absence of sensory stimuli.^10^ In the context of tinnitus, this translates into greater neuronal activity (central gain) along the auditory pathway.^11^ Although depression covaries with tinnitus prevalence and severity over time,^12^ clinically significant anxiety and stress appear as a predominant emotional comorbidity.^13^ Limbic structures have been implicated in tinnitus in humans^14,15^ and in animal models,^16^ suggesting that central regions involved in emotional processing and cognition could contribute to tinnitus^17^ and more potentially to its severity.

For decades, it has been widely believed that tinnitus is a consequence of environmental factors (as opposed to genetic factors, reviewed by Vona et al^18^). One familial aggregations study^19^ found no obvious correlation in siblings (0.16%); genotype candidate genes in patients with tinnitus failed to reveal positive associations,^20,21,22,23,24^ and a recent genome-wide association study^25^ with a small sample size found no significant associations. A twin study^26^ revealed a heritability of 0.40 based on self-reported tinnitus. It has been proposed that the lack of evidence on a significant association with genetic factors is attributable to the large heterogeneity of tinnitus and that tinnitus should not be considered a single entity but an ensemble of multiple subtypes.^27^ In support of this hypothesis, Maas et al^28^ recently found that specific forms of tinnitus had greater heritability in a sex-specific manner. When considering tinnitus perceived in 2 ears (bilateral), heritability reached 0.41 in women and 0.68 in men.^28^ In contrast, when tinnitus was heard in only 1 ear (unilateral), heritability decreased to near 0.27. However, 3 major limitations appear from these reports: (1) the sparse data could be attributable to tinnitus being self-reported and prevalence varying depending on how the question is formulated^29^; (2) self reporting tinnitus could be affected by shared-environment mechanisms whereby, for instance, a sibling with tinnitus may influence the awareness of tinnitus in the other or because both siblings live in a noisy environment; and (3) these studies did not consider tinnitus severity, which could also be associated with genetic factors, as is the case for other emotional processing disorders.^30^

We addressed these issues by performing an adoption study using national medical registry data (ie, tinnitus has been diagnosed by a physician) to determine whether shared-environment mechanisms are associated with the co-occurrence of tinnitus within a family and thus constitute a bias in the estimates of heritability.

## Methods

Data were collected on adoptees and their biological and adoptive parents from January 1, 1964, to December 31, 2015, to determine the heritability of tinnitus. Informed consent was waived as a requirement by the Ethics Committee at Lund University. Accordingly, all data were provided by Statistics Sweden and the National Board of Health and Welfare for research purposes. Data were coded according to European Union law. This study was approved by the Regional Ethical Review Board of Lund University.

We used several Swedish nationwide registers as part of our analyses. Statistics Sweden and the National Board of Health and Welfare maintain the registers used in the present study.^31,32,33,34^ The Swedish personal identity number is issued to all residents in Sweden and was used to connect individual-level data from different registers.^35^ The personal identity numbers were replaced by Statistics Sweden with serial numbers to preserve anonymity. We used data from the Swedish Multi-Generation Register, the National Patient Register (NPR), the Total Population Register, and Small Area Market Statistics (SAMS).

The Swedish Multi-Generation Register contains data on familial relationships, including adoptions. This register comprises data on index persons registered in Sweden after 1961 and born during and later than 1932.^31^ The NPR^33^ contains all hospital discharge diagnoses for all people in Sweden from 1964 to 2015. The hospital discharge register has nationwide coverage since 1987. The NPR also includes the Hospital Outpatient Register, which contains information on diagnoses from all hospital outpatient visits in Sweden between 2001 and 2015. The Primary Healthcare Register, which contains data from 1989 to 2016, was also used.^36^ The Total Population Register contains data on life events, including birth, death, name change, marital status, family relationships, educational attainment, and migration within Sweden as well as immigration to and emigration from other countries.^32^ Nearly 100% of births and deaths, 95% of immigrations, and 91% of emigrations are reported to the Total Population Register. Starting in 1991, SAMS data were used to define a municipal subarea and characterize a neighborhood; the code is composed of the county, municipality, and unique SAMS area (9200 in the whole of Sweden).

### Definition of Tinnitus and Comorbidities

Patients with tinnitus in the Swedish Hospital Discharge Register (1964-2015), Outpatient Register (2001-2015), and Primary Healthcare Register (1997-2015) were identified by the following *International Classification of Diseases* (*ICD*) codes: *ICD-7* code 781.32, *ICD-8* code 781.31, *ICD-9* code 388D, and *ICD-10* code H931. There was no primary care code that is specific for tinnitus before 1997. The main and all secondary diagnoses were used. The validity in the Hospital Discharge Register is generally between 85% and 95%.^33^

Depression, anxiety, and hearing loss were identified by *ICD* codes any time during the 1964 to 2015 follow-up period using the Swedish Hospital Discharge Register (1964-2015), Outpatient Register (2001-2015), and Primary Healthcare Register (1997-2015). *ICD* codes are presented in the eTable in the Supplement.

### Sample

The analyses were based on a data set that encompasses all Swedish-born adoptees (born between 1960 and 1990) and their biological and adoptive parents. Adoptees were excluded from the study if they had died before 16 years of age (ie, exclusion of adoptees with possible severe congenital diseases or confounding sociodemographic factors that cause children to be placed in adoptive homes), had migrated from Sweden before 16 years of age, had died before 1964 (ie, before start of follow-up), or were not linked to at least 1 biological and at least 1 adoptive parent.

All adoptees who had cohabited with a biological parent were excluded according to census data (every fifth year from 1960 to 1990) or SAMS data (yearly from 1991). Adoptees who had lived with their biological grandparent, aunt and/or uncle, and sibling or with stepparents together with their biological parent were also excluded. A total of 11 060 Swedish-born adoptees remained in the study after exclusions. They compose the study population in the cohort study. These adoptees could be linked to 19 015 adoptive parents and 17 025 biological parents. After exclusions, we identified 1029 patients (2.2%) with tinnitus among adoptees and their adoptive and biological parents. Of the 1029 cases of tinnitus, 214 were found in adoptees, 371 in biological parents, and 444 in adoptive parents.

Of the 1029 patients with tinnitus, 525 (51.0%) were found in the Outpatient Register, 23 (2.2%) in the Hospital Discharge Register, and 481 (46.7%) in the Primary Healthcare Register through *ICD* codes. Seven tinnitus cases (0.7%) were identified with *ICD-8* codes and 9 (0.9%) with *ICD-9* codes. No tinnitus case was identified with *ICD-7*. A total of 1013 tinnitus cases (98.4%) were identified with *ICD-10* codes.

Educational attainment was categorized into 4 groups: low (0–9 years), middle (10–11 years), high (≥12 years or more), and unknown.

### Statistical Analysis

We used a cohort design and a case-control approach to study genetic and nongenetic factors associated with tinnitus among adoptees. We conducted 2 main analyses. Odds ratios (ORs) were determined with logistic regression for adoptees with an affected biological parent and for adoptees with an affected adoptive parent. We used a case-control matching method (1:4) for sex, educational attainment, county of birth, and ±1 year for birth year by drawing a sample of tinnitus-affected adoptees as patients and matched control groups of tinnitus-unaffected adoptees.^37,38^ In the case-control study, we connected both groups to their biological and adoptive parents, and ORs were calculated with conditional logistic regression.

In the cohort study, logistic regression was used to determine crude (crude [univariate] models for each variable; model 1) and multivariate (adjusted model; model 2) ORs for history of tinnitus in biological or adoptive parents. In the multivariate model (adjusted model 2), we used adoptees’ birth year, sex, educational attainment, and county of birth as covariates in the cohort study. The primary outcome was OR of tinnitus in adoptees with at least 1 affected biological parent compared with adoptees without any affected biological parent. The secondary outcome was OR in adoptees with at least 1 affected adoptive parent compared with adoptees without any affected adoptive parent.

An important question in medicine is whether an observed variation in a particular disease is associated with environmental factors or biological factors (nature vs nurture debate). In genetics, heritability summarizes how heritable a disease of interest is, that is, the proportion of variance that emerges because of hereditary factors, especially with reference to the resemblance of offspring and parents.

Formally, heritability was defined as a ratio of variances, that is, the proportion of total variance that is associated with variation in additive genetic factors. According to classic quantitative genetics, the heritability of a binary trait (or disease) could be estimated by Falconer regression or with relatives’ tetrachoric correlation by presuming a liability threshold model of the disease in which everyone has a liability to develop the disease but only individuals above a threshold value do so.^39,40,41^

To evaluate heritability for tinnitus, 2 different methods were used. First, we used Falconer regression, which is based on the liability of the threshold, to obtain heritability in adoptees of the biological parents. The method and its application are described in detail by Falconer and MacKay.^40,41^ With use of the prevalence rate of the relatives of the biological probands and the controls (ie, biological parents to affected and unaffected adoptees, respectively) from the case-control study, the mean (SE) heritability was calculated. Second, we used the approach described by Frisell et al,^39^ which used the tetrachoric correlation. This method allowed us to test the sensitivity of the calculated heritability to the assumed prevalence. The tetrachoric correlation is the inferred Pearson correlation from a 2 × 2 table with dichotomous normality being assumed. We used χ^2^ and Wald tests (in logistic regression). A 2-sided *P* < .05 was considered to be statistically significant. Statistical analysis was performed with SAS software, version 9.3 (SAS Institute Inc).

## Results

We identified 1029 patients (2.2%) with tinnitus (440 [42.8%] male; mean [SD] age, 62 [14] years) in the study population during the study period (1964-2015). Table 1 gives the descriptive statistics of the adopted offspring, their biological parents, and their adoptive parents: age, sex, educational attainment, tinnitus, age at tinnitus diagnosis, and age at end of the study period (death, emigration, or end of the study period on December 31, 2015, whichever came first). The Figure shows the age distribution for Swedish-born (1960-1990) adoptees at first-time diagnosis of tinnitus. The adoptive parents, with a median age of 78 years (interquartile range [IQR], 70-83 years), were older than the biological parents, with a median age of 69 years (IQR, 59-74 years) at the end of the study period. Table 1 reports that the median birth years were 1965 (IQR, 1962-1970) for adoptees, 1942 (IQR, 1936-1947) for biological parents, and 1932 (IQR, 1927-1939) for adoptive parents. Tinnitus was found in 371 of 17 025 biological parents (2.18%) and 444 of 19 015 adoptive parents (2.33%). No statistically significant differences were found between these groups (*x^2^* = 0.99, *P* = .32). Tinnitus in adoptees, biological parents, and adoptive parents was found to be associated with depression, anxiety, and hearing loss (Table 2). The ORs were 1.89 (95% CI, 1.40-2.55) for depression, 2.19 (95% CI, 1.67-2.88) for anxiety, and 24.38 (95% CI, 18.08-32.88) for hearing loss using a crude model.

**Table 1.** Characteristics of the Study Population of Swedish-Born Adoptees Between 1960 and 1990 and Their Adoptive and Biological Parents^a^

Characteristic	Adopted Offspring (n = 11 060)	Adoptive Parents (n = 19 015)	Biological Parents (n = 17 025)
Female	5213 (47.13)	9505 (49.99)	10 358 (60.84)
Age at end of follow-up, median (IQR), y	50 (44-52)	78 (70-83)	69 (59-74)
Birth year			
Mean (SD)	1967 (7)	1933 (9)	1941 (10)
Median (IQR)	1965 (1962-1970)	1932 (1927-1939)	1942 (1936-1947)
Range	1960-1990	1888-1972	1898-1974
High educational attainment (≥12 y)	3450 (31.19)	4202 (22.10)	1452 (8.37)
Tinnitus cases	214 (1.93)	444 (2.33)	371 (2.18)
Female patients with tinnitus	113 (52.80)	244 (54.95)	232 (62.53)
Age at tinnitus diagnosis, median (IQR), y	43 (38-47)	71 (63-77)	65 (60-70)

Abbreviation: IQR, interquartile range.

^a^ Data are presented as number (percentage) of population unless otherwise indicated. The numbers of parents represent unique individuals. Some parents had several biological or adoptive children.

**Figure.**
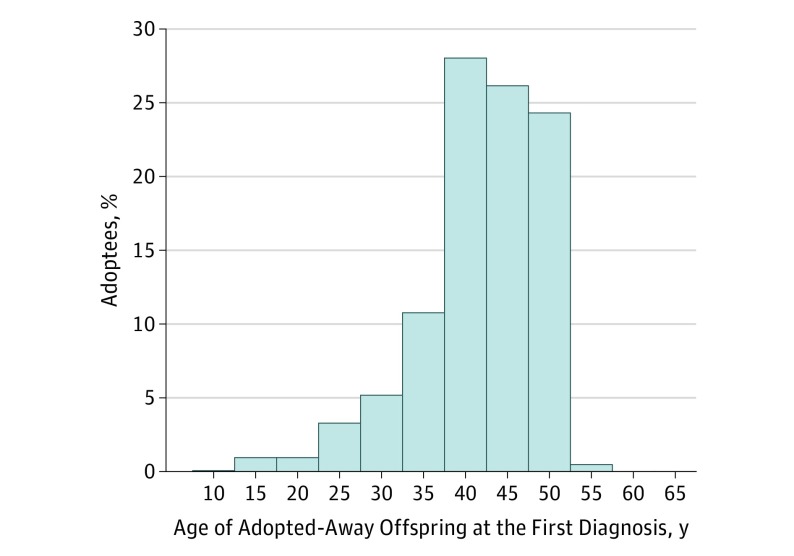
Age Distribution for Swedish-Born (1960-1990) Adoptees When Tinnitus Was First Diagnosed

**Table 2.** Prevalence of Hearing Loss, Depression, and Anxiety Among Study Participants With and Without Tinnitus Any Time During Follow-up (1964-2015)

Variable	No. (%) of Study Participants		*P* Value^a^
	No Tinnitus	Tinnitus	
**Adoptees**			
No.	10 846	214	NA
Depression	1927 (17.8)	62 (30.0)	<.001
Anxiety	2938 (27.1)	97 (45.3)	<.001
Hearing loss	310 (2.9)	97 (45.3)	<.001
**Adoptive Parents**			
No.	18 571	444	NA
Depression	1978 (10.6)	89 (20.0)	<.001
Anxiety	1943 (10.5)	111 (25.0)	<.001
Hearing loss	2 289 (12.3)	276 (62.2)	<.001
**Biological Parents**			
No.	16 654	371	NA
Depression	2194 (13.2)	92 (24.8)	<.001
Anxiety	3116 (18.7)	120 (32.2)	<.001
Hearing loss	1064 (6.4)	194 (52.3)	<.001

Abbreviation: NA, not applicable.

^a^ χ^2^ Test.

### Cohort Study

In crude model 1 (univariate), the OR for tinnitus in adoptees with at least 1 affected parent was increased (OR, 1.83; 95% CI, 1.04-3.24) (Table 3). In the fully adjusted model 2 (multivariate), which also included birth year, sex, county, educational attainment, depression, anxiety, and hearing loss, the familial OR for tinnitus was still significant at 2.01 (95% CI, 1.10-3.69). The estimated OR for tinnitus in adoptees with an affected adoptive parent was not statistically significant in the crude model 1 (univariate) (OR, 1.01; 95% CI, 0.53-1.91) or in the adjusted model 2 (multivariate) (OR, 1.04; 95% CI, 0.53-2.04). In a model that included the history of tinnitus in biological and adoptive parents, 10 179 individuals had no history of tinnitus in biological and adoptive parents, 498 individuals had adoptive parents only with tinnitus, 368 individuals had biological parents only with tinnitus, and 15 individuals had both biological and adoptive parents with history of tinnitus (interaction term Wald χ^2^ = 0.0011; SE = 351.6).

**Table 3.** Risk of Tinnitus in Adoptees as Determined by Affected Biological or Adoptive Parent (Cohort Study)

Variable	No. of Person-Years	Cases/Persons at Risk	Incidence Rate per 1000 Person-Years	Incidence Ratio (95% CI)	OR (95% CI)^a^	
					Model 1	Model 2
Risk of tinnitus with at least 1 affected biological parent						
Biological parents not affected^b^	495 278	201/10677	0.41 (0.35-0.46)	NA	NA	NA
Biological parent affected	18 122	13/383	0.72 (0.42-1.24)	1.77 (1.01-3.10)	1.83 (1.04-3.24)	2.01 (1.10-3.69)
Risk of tinnitus with at least 1 affected adoptive parent						
Adoptive parents not affected^b^	490 606	204/10 547	0.42 (0.36-0.48)	NA	NA	NA
Adoptive parent affected	22 794	10/513	0.44 (0.24-0.82)	1.06 (0.56-1.99)	1.01 (0.53-1.91)	1.04 (0.53-2.04)

Abbreviations: NA, not applicable; OR, odds ratio.

^a^ The ORs were derived from unconditional logistic regression. Model 1 is a crude model (univariate). Model 2 is an adjusted model (multivariate), with adjustments for adoptees’ birth year, sex, county, educational attainment, depression, anxiety, and hearing loss.

^b^ Reference group.

### Case-Control Study

We further validated these findings using a case-control study. Tinnitus in adoptees was significantly associated with tinnitus in biological parents, with an OR of 2.22 (95% CI, 1.03–4.81) for the 10 adoptees with an affected biological parent compared with the 136 adoptees with unaffected biological parents. Tinnitus in an adoptive parent was not significantly associated with tinnitus in adoptees (7 adoptees with an affected adoptive parent vs 139 with no affected adoptive parent; OR, 1.00; 95% CI, 0.43–2.32). These findings suggest that genetic factors are associated with the transmission of clinically significant tinnitus and that there is no association of shared environment with the transmission of tinnitus.

### Heritability

Heritability was determined in the case-control study with different estimates of the prevalence of tinnitus (Table 4). The prevalence in the particular source population is unknown; however, on the basis of a previous systematic review,^29^ a range of likely estimates was selected. The corresponding range of heritability estimates is presented in Table 4. The heritability varied from 19% in a population with 0.01% prevalence to 35% in a population with 5% prevalence. With a prevalence of 2.0% (Table 4) in the present population, the mean (SE) heritability was 31% (14%). This finding is similar to the heritability obtained using Falconer regression. The mean (SE) heritability determined using Falconer regression was 32.3% (15.7%).

**Table 4.** Heritability of Tinnitus Based on Different Estimated Population Prevalence and Tetrachoric Correlation in the Case-Control Study^a^

Prevalence	Tetrachoric Correlation, Mean (SE)	Heritability, Mean (SE), %
0.01	0.094 (0.04)	19 (8)
0.05	0.105 (0.05)	21 (10)
0.1	0.11 (0.05)	22 (10)
0.5	0.13 (0.06)	26 (12)
1.0	0.14 (0.06)	28 (12)
2.0	0.153 (0.07)	31 (14)
5.0	0.174 (0.08)	35 (16)

Abbreviation: OR, odd ratio.

^a^ Based on Frisell et al.^39^ There were 10 exposed patients (adoptees with tinnitus and an affected biological parent) and 136 unexposed patients (adoptees with tinnitus without an affected adoptive parent). The OR was 2.22 (95% CI, 1.03-4.81) for all.

## Discussion

This study identified an association between tinnitus and adoptees in relation to their biological parents but not to their adoptive parents. In other words, tinnitus in adoptive parents did not increase the odds of tinnitus among adoptees, a finding that suggests a limited association of family-related environmental factors with the heritability of tinnitus. Adoption studies are complementary to twin studies for a number of reasons. It is assumed in twin studies that concordance rates between monozygotic and dizygotic twins are comparable and can thus be used to estimate genetic contributions because the twins share the same environment. However, one study^42^ suggests that monozygotic twins are treated more equally than dizygotic twins, which in theory would inflate the heritability seen in twin studies. Whether violation of the equal environments assumption in twin studies for tinnitus affects estimates of heritability is not known. It is also not known whether the assumption of random mating affects the estimates of tinnitus heritability. Because adoptees do not grow up in their biological families, adoption studies offer an excellent model to investigate genetic influences of a given trait. Transmission from adoptive parents to nonbiological offspring would be mainly associated with environmental factors, whereas the transmission from biological parents to offspring would therefore be associated with genetic factors. However, although this notion is important to understand the transmission of tinnitus, the present work cannot completely rule out the contribution of shared environment in tinnitus development. In this study, most adoptees were diagnosed with tinnitus in adulthood, and whether familial environmental factors are weakened or not after adoptees become adults remains uncertain. Familial aggregation studies, including influence on spouses, may help complement the present study; however, one should consider that different mechanisms of development of severe tinnitus may occur between men and women, as recently suggested.^13^

### Strengths and Limitations

A major strength in the present study is the use of nationwide registers, which are almost complete and have successfully been used to estimate familial risks for a number of diseases.^31,32,33,34,36,43^ Moreover, the study design eliminates the risk of recall bias, which is an important problem in case-control studies. We reveal a heritability of 32%, which is in the range of the values that 2 former Swedish twin studies reported (near 40% for any type of tinnitus).^26,28^ The study from Bogo et al^26^ used a sample of twins (n = 1084 individuals) whose pure-tone audiometric thresholds (up to 8 kHz) were available, whereas the study from Maas et al^28^ estimated heritability on a larger sample size (n = 10 464 twin pairs) in the absence of audiometric data. These 2 twin studies^26,28^ relied on self-reported tinnitus, which does not specify the degree of severity, the duration (acute vs chronic), and the time component (occasional vs permanent). In addition, the way that the question was formulated may cause substantial differences in the reported prevalence,^29^ which in turn can affect heritability values. In this study, heritability values were obtained using medical registries based on a diagnostic established by a physician, which may be seen as a more rigorous approach, although not free from biases. The prevalence of 2% appears on the low side of the mean prevalence of self-reported severe tinnitus in the literature.^29^ The number of people with tinnitus seeking help may be lower in this study because of the general lack of trust in the ability of physicians to provide a solution to their tinnitus.^44^ Because heritability values are influenced by prevalence, the values obtained in our study were potentially underestimated. Tinnitus assessment and treatment are not standardized at the national level in Sweden; however, the Stockholm County Council has developed a document to guide the assessment of tinnitus at the general practitioner level using the Tinnitus Handicap Inventory and proposed some rehabilitation options, such as cognitive behavioral therapy and a modified tinnitus retraining therapy. A patient is directed for special rehabilitation when the Tinnitus Handicap Inventory score is 58 or higher (meaning severe or catastrophic) or when clinically significant levels of anxiety or depression are found. How tinnitus assessment and rehabilitation are performed in other counties is unclear, and therefore the global picture of tinnitus care in Sweden is missing. Thus, the country would benefit from national tinnitus guidelines,^45^ which would harmonize the reporting procedures and national registry data. The problem of the clinical assessment of tinnitus is not restricted to Sweden because of the disparity in assessing tinnitus if found across all European Union countries (Cederroth et al, unpublished data, 2017), which emphasizes the need to standardize its clinical assessment until objective tools can be implemented in the health care system.

Assuming that occasional tinnitus precedes the development of permanent tinnitus and mild tinnitus also precedes the development of severe tinnitus would mean that the current study using medical registries, in which a diagnosis is reported, would underestimate the heritability values by focusing only on clinically significant tinnitus. A focus on severe tinnitus may reveal heritability values that would not be directly associated with the development of tinnitus per se; instead, these values would be more associated with the accompanying psychological distress. Whereas none of the investigated polymorphisms have been associated with tinnitus (using a group without tinnitus as controls), a variant in the promoter of the serotonin transporter gene (*SLC6A4*) (OMIM 182138) was associated with tinnitus severity when compared with patients with milder tinnitus.^46^ Similarly, a pilot genome-wide association study^25^ with a small sample size of 167 patients and 769 controls found no significant associations but revealed an enrichment in genes involved in serotonin receptor signaling. These studies are consistent with recent findings in mice whereby serotonin would excite fusiform cells from the dorsal cochlear nucleus,^47^ a structure from the auditory pathway known to be involved in tinnitus.^35,48,49,50^ Thus, the heritability values collected in our study may reflect the genetic heritability of severe tinnitus and not that of any type of tinnitus.

A study from Martinez et al^51^ suggests that the existing *ICD* codes are of sufficient quality for research in a clinical setting. However, we believe that the existing *ICD* codes for tinnitus are obsolete, even when considering *ICD* codes for hearing loss, and would still benefit from a global revision. Laterality (meaning whether tinnitus is perceived unilaterally or bilaterally) appears as an important classification for genetic subtypes of tinnitus according to a recent twin study.^28^ Maas et al^28^ found that bilateral tinnitus is significantly influenced by genetic factors, whereas unilateral tinnitus is more subject to environmental influences. However, the degree of hearing loss in patients with unilateral and bilateral tinnitus appears to be similar (Cederroth et al, unpublished data, 2018), and thus the establishment of laterality using audiologic register data may not help in distinguishing patients with bilateral from those with unilateral tinnitus. Furthermore, although hearing loss and noise exposure are major etiologic factors for tinnitus, a nonnegligible proportion of patients experience tinnitus without hearing loss, supporting the notion that the current codes for tinnitus or hearing loss would not facilitate the replication of the study by Maas et al.^8^ A revision of the *ICD* codes for tinnitus is an endeavor that will require substantial evidence to classify tinnitus subtypes according to clear criteria. Fortunately, temporary *ICD* codes can be generated for research purposes at a national level and may help determine whether a newly proposed definition has clinical value before its implementation into the World Health Organization codes. Nevertheless, the consistency among these 3 studies in Sweden supports the idea that some forms of tinnitus are associated with genetics more than with shared environment. How this applies to other countries needs to be determined using similar approaches.

Although it is likely that most adoptions occurred in early childhood, we lack information regarding the age at which children were adopted. Perinatal factors and preplacement age could confound the genetic component. In previous studies,^52^ most children were adopted before 12 months of age. Another limitation is demographic differences between biological and adoptive parents. For instance, biological parents (including biological parents with tinnitus) were more often women than adoptive parents. Biological parents (including biological parents with tinnitus) were also younger than adoptive parents. In addition, the present adoption study included only adoptees who were born in Sweden; therefore, we cannot generalize the present study to a population of nonwhite origin.

## Conclusions

This adoption study using national registry data suggests that clinically significant tinnitus is associated with genetic factors, with a heritability of 32% and that there is no association between shared-environment factors with the transmission of tinnitus. Because patients in Sweden with a diagnosis of tinnitus most often have severe tinnitus, the present data reveal that there could be an association between genetic factors and the transition from compensated to decompensated tinnitus. Thus, the association of genetics with tinnitus might be found on 2 levels: (1) for determining whether an individual develops tinnitus or not and (2) for determining whether an individual transitions from having nonbothersome tinnitus to bothersome tinnitus. The identification of genes involved in any of these 2 aspects may provide interesting insights into the molecular mechanisms that regulate phantom percepts and their treatment.

## Notes

Supplement. eTable. International Classification of Diseases (ICD) Codes Used to Define Depression, Anxiety, and Hearing Loss
