# Supplementary material for: Association of Genetic vs Environmental Factors in Swedish Adoptees With Clinically Significant Tinnitus
Source: JAMA Otolaryngol Head Neck Surg. 2019 Jan 17;145(3):222–9. doi: 10.1001/jamaoto.2018.3852 (PMC6439751; doi:10.1001/jamaoto.2018.3852)
Supplement: Supplement. — eTable. International Classification of Diseases (ICD) Codes Used to Define Depression, Anxiety, and Hearing Loss [file jamaotolaryngolheadnecksurg-145-222-s001.pdf]

Supplementary Online Content

Cederroth CR, Fard MP, Trpchevska N, et al. Association of genetic vs environmental factors in Swedish adoptees with clinically significant tinnitus. *JAMA Otolaryngol.* Published online January 17, 2019. doi:10.1001/jamaoto.2018.3852

eTable. International Classification of Diseases (ICD) Codes Used to Define Depression, Anxiety, and Hearing Loss

This supplementary material has been provided by the authors to give readers additional information about their work.

© 2018 American Medical Association. All rights reserved.

eTable. International Classification of Diseases (ICD) Codes Used to Define Depression, Anxiety, and Hearing Loss

	ICD codes for the Swedish Hospital Discharge Register and Outpatient Register				ICD codes for Primary health care register	
	ICD-10	ICD-9	ICD-8	ICD-7	1997-2015	1987-1996
Depression	F30-F39	296	296	301, 302.99	F30-F39	296
Anxiety	F40-F48	300	300	310-318	F40-F48	300
Hearing loss	H80, H810, H833, H90, H91	386A, 387, 388C, 388B, 389	385.99, 386,99, 388, 389	395.99, 396.00, 397, 398	H80, H810, H833, H911, H919	387

© 2018 American Medical Association. All rights reserved.
